# The Rise in Carbapenem-Resistant *Acinetobacter baumannii* and the Emergence of Eravacycline as a Treatment Strategy: A Narrative Review

**DOI:** 10.3390/pathogens15060642

**Published:** 2026-06-16

**Authors:** Bo Guan, Le Zhang, Chunling Zhang, Jing Huang

**Affiliations:** 1 Department of Pharmacy, The Second Affiliated Hospital of Air Force Medical University, Xi’an 710038, China; 2 Department of Clinical Pharmacy, Honghui Hospital, Xi’an Jiaotong University, Xi’an 710054, China

**Keywords:** *Acinetobacter baumannii*, eravacycline, multidrug resistance, resistance mechanism, narrative review

## Abstract

*Acinetobacter baumannii* is a significant pathogen of hospital-acquired infections, and its multidrug resistance (MDR) and extended drug resistance (XDR) have become increasingly severe, posing a global public health challenge. This article provides a narrative review of the major resistance mechanisms of *Acinetobacter baumannii*, including β-lactamase production, efflux pump overexpression, target site modification, reduced membrane permeability, and biofilm formation. Additionally, it summarizes the current main drugs and their target sites for treating MDR *Acinetobacter baumannii* infections, with a focus on the mechanism of action, antibacterial activity, and clinical research progress of the novel fully synthetic fluorocycline antibiotic—eravacycline. Eravacycline inhibits protein synthesis by high-affinity binding to the bacterial ribosomal 30S subunit and demonstrates activity against multidrug-resistant *Acinetobacter baumannii* (excluding *Pseudomonas aeruginosa*), providing a potential novel therapeutic option for MDR/XDR *Acinetobacter baumannii* infections. Finally, the article outlines future research directions and treatment strategies. Due to the narrative nature of this review, no systematic methodology (e.g., PRISMA) was applied, and the available clinical evidence, particularly for CRAB infections, remains limited.

## 1. Introduction

As a non-fermenting Gram-negative bacillus, *Acinetobacter baumannii* is widely distributed in hospital environments and can cause various severe conditions such as pneumonia, bloodstream infections, urinary tract infections, and wound infections. In recent years, the detection rate of carbapenem-resistant *Acinetobacter baumannii* (CRAB) has been continuously increasing worldwide, leading to a significant rise in clinical treatment failure rates and patient mortality. The drug resistance mechanisms of this bacterium are complex, often presenting as multidrug resistance (MDR), resulting in a severely limited range of available antimicrobial agents.

Carbapenem-resistant Gram-negative bacilli encompass CRAB and carbapenem-resistant *Enterobacteriaceae* (CRE), including carbapenem-resistant *Klebsiella pneumoniae* (CRKP) and carbapenem-resistant *Pseudomonas aeruginosa* (CRPA). Globally, CRAB prevalence varies considerably by region. According to data from the European Centre for Disease Prevention and Control (ECDC), the proportion of carbapenem-resistant *Acinetobacter* species among invasive isolates exceeds 50% in several Southern and Eastern European countries. In the United States, the Centers for Disease Control and Prevention (CDC) has identified CRAB as an urgent threat, with approximately 8500 cases reported annually in hospitalized patients [1,2]. In China, the prevalence of CRAB has shown an upward trend, with the detection rate reaching as high as 73.7% in 2023. Reports on CRAB infections in hospitalized patients have increased, with an incidence rate ranging from 8.2% to 50.0% and a mortality rate as high as 80%. Common infection types include hospital-acquired pneumonia (HAP), ventilator-associated pneumonia, bacteremia, urinary tract infections, and skin and soft tissue infections [3]. CRAB bacteremia is a severe infectious complication following liver transplantation, with risk factors for CRAB infection within 30 days postoperatively including pre-transplant end-stage liver disease model (MELD) score, severe encephalopathy, donor body mass index, and reoperation [4].

Given the urgent need for novel therapeutic options against MDR/XDR *Acinetobacter baumannii*, this article aims to provide a narrative review of the resistance mechanisms of *Acinetobacter baumannii* and existing treatment strategies, with a focus on investigating the pharmacological characteristics and clinical value of the novel fluorocycline antibiotic eravacycline.

## 2. Resistance Mechanisms and Current Treatment Strategies for *Acinetobacter baumannii*

### 2.1. Major Resistance Mechanisms

The drug resistance of *Acinetobacter baumannii* results from the synergistic action of multiple mechanisms, primarily encompassing the following aspects:

Production of antimicrobial inactivating enzymes: The generation of various β-lactamases is one of the primary resistance mechanisms. In addition to the commonly encountered extended-spectrum β-lactamases (ESBLs) and AmpC enzymes, the prevalence of carbapenemases (particularly OXA-type enzymes) is a key factor contributing to carbapenem resistance. Furthermore, aminoglycoside-modifying enzymes (AMEs) also mediate high-level resistance to aminoglycoside drugs [5].

Overexpression of drug efflux pump systems: *Acinetobacter baumannii* possesses multiple efflux pump systems of the resistance–nodulation–division (RND) family, such as AdeABC, AdeFGH, and AdeIJK. Among these, the overexpression of the AdeABC system is associated with resistance to various drugs (including β-lactams, fluoroquinolones, tetracyclines, tigecycline, and aminoglycosides), representing a key mechanism underlying multidrug resistance [6].

Alteration of drug action targets: Changes in penicillin-binding proteins (PBPs) or the expression of low-affinity PBPs can reduce the binding efficiency of β-lactam antibiotics. Gene mutations in DNA gyrase (GyrA/B) and topoisomerase IV (ParC/E) lead to fluoroquinolone resistance. The production of 16S rRNA methyltransferases (e.g., ArmA, RmtB) enables bacteria to develop high-level resistance to all aminoglycoside antibiotics.

Decreased membrane permeability: The deletion or downregulation of outer membrane pore proteins (e.g., CarO, OprD-like) reduces the entry of antimicrobial agents into the bacterial cell. Structural modifications of lipopolysaccharide (LPS) may also enhance the stability of the bacterial outer membrane, further limiting drug permeation.

Formation of biofilms: Biofilms provide bacteria with a physical barrier, significantly reducing the permeability of antimicrobial agents and inducing them into a metabolic dormancy state, thereby evading the lethal effects of antimicrobial drugs.

Horizontal transmission of resistance genes: Mobile genetic elements such as plasmids, transposons, and integrons rapidly disseminate resistance genes among different strains of *Acinetobacter baumannii* and even across different species, accelerating the spread of drug resistance [7].

Critical Analysis: Which Resistance Mechanisms Are Most Important in CRAB Today?

Among the mechanisms described above, OXA-type carbapenemases (particularly OXA-23, OXA-24/40, and OXA-58) are currently the most clinically significant drivers of carbapenem resistance in *Acinetobacter baumannii* worldwide. These enzymes hydrolyze carbapenems with high efficiency and are often encoded on mobile genetic elements, facilitating rapid spread. The AdeABC efflux pump represents the second most important mechanism, as its overexpression confers resistance not only to carbapenems but also to multiple other antibiotic classes, including tetracyclines (e.g., tigecycline) and aminoglycosides. In contrast, reduced membrane permeability and biofilm formation, while contributory, are generally considered secondary or supportive mechanisms that enhance resistance but rarely act as primary drivers.

### 2.2. Current Therapeutic Agents and Strategies

For clinical management of MDR/XDR *Acinetobacter baumannii* infections, treatment regimens are typically determined based on antimicrobial susceptibility testing results, employing combination therapies to enhance therapeutic efficacy and prevent the development of resistance [5].

Commonly Used Antimicrobial Agents and Their Targets

Polymyxins (colistin, polymyxin B): Their target is bacterial lipopolysaccharide, serving as the “last line of defense” for treating CRAB. However, monotherapy is prone to inducing heterogeneous resistance and carries a high risk of nephrotoxicity and neurotoxicity.

Tigecycline: The target of action is the 30S subunit of the ribosome. This drug exhibits certain in vitro activity against CRAB, but its plasma concentration is low, resulting in limited efficacy in treating pneumonia and bloodstream infections. It is not recommended for monotherapy.

Sulbactam and its combination preparations: In addition to inhibiting certain β-lactamases, sulbactam exhibits unique affinity for penicillin-binding protein 2 (PBP2) of *Acinetobacter baumannii*. High-dose ampicillin–sulbactam or cefoperazone–sulbactam are among the commonly used therapeutic options.

Carbapenems: Only effective against certain susceptible or intermediate strains. For CRAB, high-dose, prolonged infusion of carbapenems combined with other agents may exhibit a certain synergistic effect.

Novel enzyme inhibitor combinations: Agents such as ceftazidime–avibactam are effective against certain serine carbapenemases (e.g., KPC, OXA-48) but ineffective against common OXA-23 enzymes in *Acinetobacter baumannii*, thus limiting their clinical application.

Combination Therapy Strategies: The combination of two or three drugs is the mainstream treatment strategy. Common drug combinations include: polymyxin-based regimens combined with rifampin, carbapenems, or tigecycline; and sulbactam-based formulations combined with polymyxin or tigecycline. The goal of combination therapy is to achieve synergistic effects and reduce the selection window for drug-resistant mutations [8].

### 2.3. Novel Therapeutic Agents for CRAB

Recent years have seen the approval and development of several novel agents with activity against CRAB. Cefiderocol, a siderophore cephalosporin, utilizes bacterial iron uptake systems to achieve high periplasmic concentrations and has demonstrated in vitro activity against CRAB, although clinical efficacy data remain mixed. Sulbactam-durlobactam is a novel β-lactam–β-lactamase inhibitor combination specifically developed for *Acinetobacter*; durlobactam protects sulbactam from degradation by OXA-type carbapenemases, and the combination has shown promising results in clinical trials. These agents, along with eravacycline, represent emerging therapeutic options, though comparative effectiveness data are still limited.

## 3. Eravacycline: Mechanism of Action and Pharmacological Properties

### Drug Structure and Target

Eravacycline, as a new-generation fully synthetic fluorocycline antibiotic belonging to the tetracycline class, was approved by the FDA in 2018 under the brand name Xerava^®^, primarily for the treatment of complicated intra-abdominal infections (cIAI). In 2020, the Infectious Diseases Society of America (IDSA) issued guidelines for treating drug-resistant Gram-negative bacterial infections, recommending eravacycline as a treatment option for infections caused by carbapenem-resistant *Enterobacteriaceae*. In 2023, its intravenous formulation was approved for marketing in China, with indications and usage consistent with the aforementioned.

The structure of eravacycline consists of a tetracycline core with two unique modifications: the introduction of a fluorine atom at the C7 position of the D-ring and the addition of a pyrrolidinylacetamide group at the C9 position. Given these specific modifications at the C7 and C9 positions, eravacycline has demonstrated therapeutic effects in laboratory studies against certain Gram-positive and Gram-negative bacteria with tetracycline-specific acquired resistance mechanisms [9]. The molecular structure, featuring a fluorine atom at C7 and a pyrrolidinylacetamide group at C9, not only significantly enhances antibacterial activity but also optimizes drug permeability, tissue distribution, and metabolic stability [10].

Similar to other tetracycline drugs, eravacycline exerts its effect by blocking bacterial protein synthesis [11]. It has high antibacterial activity against Gram-negative and Gram-positive aerobic, facultative anaerobic, and obligate anaerobic bacteria, as well as their drug-resistant strains. In vitro studies have shown that eravacycline has a tenfold higher affinity for binding to the ribosome than tetracycline and can inhibit protein translation at a concentration that is only a quarter of that of tetracycline. Although tetracycline drugs, including eravacycline, are usually bacteriostatic, eravacycline has shown bactericidal activity against specific strains of *Klebsiella pneumoniae*, *Escherichia coli*, and *Acinetobacter baumannii* in vitro experiments [12].

Important note: Direct comparative data on drug accumulation, transport kinetics, or binding constants with the 30S subunit in *Acinetobacter baumannii* compared to tigecycline are currently lacking. The observed changes in MIC with adeB disruption support the role of efflux, but do not directly demonstrate superiority of the binding mechanism.

Eravacycline is one of the most potent derivatives in the fluorinated tetracycline series and can cover most common clinical pathogenic bacteria except *Pseudomonas aeruginosa*, against which its activity is limited [13]. Compared with tigecycline, some in vitro studies suggest that eravacycline may have lower MIC values against certain strains of *Acinetobacter baumannii* (2 to 4 times lower in some studies); however, clinical data directly comparing the two drugs for CRAB infections are lacking. The most common adverse reactions of eravacycline include gastrointestinal adverse reactions such as nausea, vomiting, and diarrhea, but generally do not lead to drug discontinuation [14,15,16]. Currently, eravacycline is recommended by the Infectious Diseases Society of America (IDSA) and the Chinese guidelines [17] as one of the alternative treatment options for β-lactam drug resistance. Like other tetracycline drugs, eravacycline may cause adverse reactions such as hypersensitivity and irreversible tooth discoloration [11].

## 4. In Vitro Activity of Eravacycline Against Drug-Resistant *Acinetobacter baumannii*

It is important to emphasize that the following data are derived primarily from in vitro studies. All data was summarized in Table 1. While these provide valuable information on antibacterial activity, they do not directly predict clinical efficacy, particularly for infection types such as pneumonia or bacteremia.

Interaction with efflux pumps: Eravacycline maintains good in vitro activity against *Acinetobacter baumannii* strains that overexpress RND efflux pumps such as AdeABC. Available data suggest that eravacycline may be less affected by certain RND efflux systems compared with older tetracyclines, although direct comparative studies with tigecycline in *Acinetobacter baumannii* regarding drug accumulation, transport kinetics, or ribosomal binding constants are currently lacking.

Overcoming ribosomal protection: For strains carrying ribosomal protection protein genes such as tet(M), eravacycline has demonstrated stronger antibacterial activity in vitro compared with tetracycline.

Broad-spectrum anti-enzyme activity: Eravacycline shows stable in vitro antibacterial activity against *Acinetobacter baumannii* that produces carbapenemases (OXA-type, NDM-type, etc.) and ESBLs. Multiple global surveillance data indicate that its MIC90 value against CRAB is usually ≤0.5 mg/L, and its in vitro activity appears comparable to or slightly better than that of tigecycline against certain isolates.

In vivo efficacy: In animal infection models, eravacycline has shown therapeutic effects in pneumonia and bloodstream infection models. Its tissue distribution is extensive, with higher concentrations reported in lung tissue and abdominal cavity compared with plasma.

A study compared the in vitro susceptibility of 275 MDR Gram-negative clinical isolates collected continuously by the Belgian National Research Council (NRC). The test strains included species from the *Enterobacteriaceae* family (n = 222) and *Acinetobacter* genus (n = 53), with the majority being carbapenemase-producing bacteria. According to the broth microdilution method, 83.3% of the *Enterobacteriaceae* were susceptible to eravacycline. For the *Acinetobacter* genus, the MIC50 of eravacycline was 0.25 mg/L; tigecycline was 0.5 mg/L; 56.6% of the *Acinetobacter* genus were susceptible to eravacycline. Eravacycline may be a potential therapeutic option for treating MDR *Enterobacteriaceae*, with an activity rate comparable to that of tigecycline and cefiderocol. For MDR *Acinetobacter*, eravacycline showed moderate in vitro activity, although its MIC50 value was one dilution lower than that of tigecycline [21].

The treatment of CRAB is facing increasingly severe clinical challenges. CRAB isolates were screened for carbapenemase genes, with the most common being blaOXA-23. The MIC50 and MIC90 of eravacycline were 0.125 μg/mL and 0.5 μg/mL, respectively. The results of this in vitro study indicate that combination therapy of eravacycline with other agents may provide promising therapeutic potential, but further clinical evaluation is needed [22].

## 5. Clinical Research and Application: Current Evidence and Limitations

### 5.1. Approved Indications and Pharmacokinetics

Currently, eravacycline has been approved by the US FDA and the EMA for the treatment of cIAI. It is important to note that eravacycline is not currently approved for the treatment of pneumonia or bloodstream infections, including those caused by CRAB. The following clinical data, unless otherwise specified, are derived from cIAI studies and may not be directly generalizable to other infection types.

The oral bioavailability of eravacycline is 28%, with an average half-life (T1/2) of 22 h, 16% renal excretion, and a protein binding rate of 79–100%. It is widely distributed in vivo, with higher exposure in lung tissue than in plasma in animal models. Animal studies indicate elevated concentrations of eravacycline in lung, kidney, liver, and bile tissues [23]. Eravacycline is primarily metabolized via CYP3A4/5 and FMO-mediated oxidation, with potential drug–drug interactions. No dose adjustment is required for patients with renal insufficiency, but dose adjustment is necessary for those with severe hepatic impairment.

### 5.2. Phase III Clinical Trials (cIAI)

A Phase III clinical trial comparing eravacycline with ertapenem 1 g once daily for the treatment of cIAI met the statistical non-inferiority criteria [24]. Another Phase III clinical trial (IGNITE 4) comparing eravacycline with meropenem 1 g every 8 h for the treatment of cIAI demonstrated that eravacycline was non-inferior to meropenem [25].

In the IGNITE4 clinical trial (NCT02784704), eravacycline demonstrated non-inferior efficacy compared to meropenem in treating patients with complicated intra-abdominal infections, with a clinical cure rate of 92.4%. It was comparable to meropenem in terms of microbiological clearance rate (90.8% vs. 91.2%), adverse event incidence rate (37.2% vs. 30.9%), and mortality rate (1.6% vs. 0.4%) [25]. However, this trial did not stratify outcomes by adequacy and timeliness of source control, a key factor in complicated intra-abdominal infections that may influence outcomes to a greater extent than antibiotic selection. Bayesian network meta-analysis indicated that the microbial response rate of eravacycline was significantly superior to that of tigecycline [26].

Crucial limitation: These trials enrolled patients with cIAI, not CRAB pneumonia or bacteremia. The pharmacokinetic/pharmacodynamic characteristics of eravacycline in lung tissue and blood, as well as its clinical efficacy for these infection types, remain incompletely defined.

### 5.3. Real-World and Retrospective Studies (CRAB Infections)

A study evaluated the real-world application of eravacycline in 13 hospitalized patients with complex infections (including some CRAB cases). The clinical cure rate was 69.2%, and the mortality rate was 38.5%. Most patients received combined treatment (mainly β-lactam antibiotics). One suspected case of drug-induced liver toxicity was observed. The research results were highly consistent with previous case series. However, due to the very small sample size (n = 13) and the retrospective design, these findings should be considered preliminary and hypothesis-generating rather than definitive. Further controlled studies are needed to clarify the role of eravacycline in complex infections [27].

A retrospective study analyzed 65 ICU patients with CRAB pneumonia from September 2023 to March 2024, comparing the clinical outcomes of eravacycline versus tigecycline as targeted therapy. The 30-day mortality rate was numerically lower in the eravacycline group (15.2% vs. 25.0%), but this difference did not reach statistical significance in multivariate analysis. Notably, no deaths occurred in the eravacycline-treated patients who received infectious disease consultation. There were no significant differences in the rates of symptom remission and cure between the two groups. The authors concluded that in the treatment of CRAB pneumonia, eravacycline demonstrated non-inferior efficacy to tigecycline and exhibited favorable safety profiles. Important caveats: This was a retrospective, non-randomized study with potential selection bias. The association between eravacycline use and lower mortality could be confounded by other factors, including the timing of therapy initiation, use of combination regimens, and involvement of infectious disease specialists [28].

### 5.4. Safety Profile

Although other tetracycline drugs (especially tigecycline) have been reported to cause pancreatitis [22,29], the incidence of adverse events (mainly gastrointestinal reactions such as nausea and vomiting) of eravacycline was higher than that of control drugs in clinical trials, though these events were generally mild to moderate. Therefore, when using eravacycline in cIAI patients or patients with elevated lipid levels, this possibility should be considered.

### 5.5. Summary of Clinical Evidence Limitations

The following limitations of the clinical evidence base for eravacycline in CRAB infections must be acknowledged: No phase III trials specifically for CRAB pneumonia or bacteremia. Most CRAB-specific clinical data come from small, retrospective studies with potential confounding. Approved indications are limited to cIAI; off-label use for CRAB pneumonia requires caution. Causality between eravacycline use and improved clinical outcomes cannot be conclusively established due to confounding variables (combination therapy, source control, specialist consultation).

## 6. Resistance to Eravacycline and Its Mechanisms

The resistance mechanisms to eravacycline are associated with mutations in 16S rRNA and tetracycline degradation genes, enhanced efflux activity, and the absence of OmpF and OmpC pore protein expression. In certain bacteria, resistance to eravacycline is linked to modifications of the target site, particularly through the overregulation of non-specific intrinsic MDR efflux pumps. In *Enterococcus* species, resistance to eravacycline has been detected, with isolates carrying mutations in the rpsJ gene. To date, no cross-resistance based on target mechanisms between eravacycline and other antibiotic classes (including carbapenems, cephalosporins, penicillins, and fluoroquinolones) has been identified [12].

In a study evaluating the in vitro efficacy of eravacycline against tetracycline-resistant *Escherichia coli* strains, no significant changes in eravacycline efficacy were observed in strains carrying tetracycline resistance genes (tetA, tetB, tetK, and tetM), or only minor changes were noted. Specifically, in strains expressing the tetA efflux pump, the minimum inhibitory concentration of eravacycline was increased by fourfold. However, this increase was significantly lower compared to the magnitude observed with tigecycline and other tetracycline antibiotics. These findings suggest that eravacycline may exhibit higher efficacy against tetracycline-resistant bacteria in vitro [30].

In vitro studies demonstrated that eravacycline exhibited significant antibacterial activity against naturally expressed mcr-1 bacterial strains (resistant to polymyxin antibiotics such as colistin) and clinically acquired CRE isolates with overexpression of mcr-1. Additionally, eravacycline showed potent antibacterial effects against polymyxin-resistant *Escherichia coli* and *Klebsiella pneumoniae* strains carrying the mcr-1 gene in laboratory settings [12,31].

Abdallah et al. identified a significant correlation between eravacycline MIC and adeB efflux gene expression in 38 *Acinetobacter baumannii* isolates (MIC range 0.06–4 mg/L) through multiple regression analysis. In isolates overexpressing the adeB gene, disruption of the adeB gene reduced the eravacycline MIC from 2 mg/L to 0.25 mg/L. Samples exhibiting typical adeB activity levels demonstrated that the eravacycline MIC remained at 0.25 mg/L after adeB gene disruption.

Regarding combination therapy, studies using gradient strip methods have suggested potential synergistic effects of eravacycline with meropenem-vaborbactam or ceftazidime against certain CRAB strains [32]. However, these findings are based on gradient strip assays, which are considered preliminary; confirmation via checkerboard or time-kill assays is required before definitive conclusions can be drawn.

Safety Considerations

The spectrum of adverse reactions is similar to that of tigecycline, with the most common being gastrointestinal reactions (nausea, vomiting), typically mild to moderate. Other serious adverse reactions occur less frequently. Eravacycline has been associated with coagulation disorders, and there have been reports of tigecycline-induced thrombocytopenia, frequent coagulation disorders, decreased fibrinogen levels, and strong signals of hypofibrinemia. In one case series, when tigecycline was replaced with eravacycline, hypofibrinemia persisted, whereas coagulation function gradually recovered after switching to omadacycline [33]. This difference may be related to the C7 fluorine substituent, as the fluorinated structure of eravacycline can inhibit fibrinogen production via the hepatic toxicity pathway characteristic of perfluorinated compounds [10]. The specific mechanism of action and the structure-activity relationship with tetracyclines require further validation.

## 7. Conclusions

The resistance situation of *Acinetobacter baumannii* is severe, and its resistance mechanisms are complex and intertwined. Current treatment relies on limited old drugs and combination regimens, and new effective weapons are urgently needed. Eravacycline, as a new generation of tetracycline derivative, has shown promising in vitro activity against MDR/XDR *Acinetobacter baumannii* and has a well-defined mechanism of action (high-affinity binding to the 30S ribosomal subunit). However, several important limitations must be recognized: Clinical evidence for CRAB infections remains limited. Most data come from cIAI trials, small retrospective studies, or animal models. Eravacycline is not currently approved for pneumonia or bloodstream infections, which are among the most common and severe CRAB infection types. The association between eravacycline use and improved clinical outcomes (e.g., reduced mortality) cannot be conclusively established due to confounding factors such as combination therapy, source control, and specialist consultation. Direct comparative data with tigecycline in CRAB infections are lacking.

Future research directions should include: conducting randomized controlled trials specifically targeting CRAB pneumonia and bloodstream infections to confirm the efficacy and safety of eravacycline; exploring the optimal combination of eravacycline with existing antibacterial drugs to maximize synergistic effects and prevent resistance; and continuously monitoring the emergence and development of eravacycline resistance.

In summary, while eravacycline represents a valuable addition to the armamentarium against MDR Gram-negative bacteria, including CRAB, its role should be viewed as a potential option rather than a proven standard of care for CRAB pneumonia or bacteremia. Clinical use should be guided by antimicrobial susceptibility testing, and combination therapy with other active agents is generally recommended until more robust clinical data become available.

## Figures and Tables

**Table 1:** Summary of In Vitro Activity of Eravacycline Against Drug-Resistant *Acinetobacter baumannii*.

Study	Strain Characteristics	MIC_50_ (mg/L)	MIC_90_ (mg/L)	Key Findings
Zhao et al. (2019) [18]	CRAB from 11 hospitals in China	0.5	1	Superior to tigecycline
Galani et al. (2023) [19]	CRAB bloodstream isolates, Greece	0.25	0.5	MIC_90_ lower than tigecycline
Seifert et al. (2018) [20]	Carbapenem-nonsusceptible *A. baumannii*	0.25	0.5	Susceptibility > 90%
Kinet-Poleur et al. (2025) [21]	MDR *Acinetobacter* spp.	0.25	-	MIC_50_ one dilution lower than tigecycline
El-Ashry et al. (2025) [22]	CRAB (OXA-23 producers)	0.125	0.5	Synergistic potential with colistin

The studies cited above include data from *Acinetobacter* spp. in some cases; species-level identification was not uniformly performed, which limits direct generalization to *Acinetobacter baumannii* alone.

## Data Availability

No new data were created or analyzed in this study.
